# Viability dataset on microencapsulated probiotics: Sodium alginate viscosity effect

**DOI:** 10.1016/j.dib.2019.104735

**Published:** 2019-11-02

**Authors:** Araceli Olivares, Paulina Silva

**Affiliations:** Centro Regional de Estudios en Alimentos Saludables (CREAS), Chile

**Keywords:** Microencapsulation, Vibration technology, Viscosity, Sodium alginate, *Lactobacillus casei*

## Abstract

Probiotics must be delivered alive to exert a positive health effects in site of action. But, they must survive different extreme condition through intestinal tract. Microencapsulation techniques have received considerable attention and facilitate a suitable carrier system to reach the target site. The encapsulation techniques applied to probiotics can be classified into two groups, depending on the method used to form the beads: extrusion (droplet method) and emulsion or two-phase system [1], where extrusion is evolved in the vibration technology and in particular, when the wavelength of an asymmetric disturbance exceeds the jet circumference, the break-up occurs. Droplet size depends on nozzle (jet) diameter, viscosity of fluid, surface tension, jet velocity and frequency of disturbance [2,3].

The data presented in this article evaluated the performance of microencapsulated *Lactobacillus casei* (probiotic bacteria) using vibration technology and using two kinds of sodium alginate gel matrix (low and medium viscosity) and compare the effect over viability.

The best conditions for higher viability of probiotics were at a concentration of sodium alginate (medium viscosity) at 2%, with a nozzle of 450 μm and a frequency of 1000 Hz. The data are related to the research article entitled “*Microencapsulation of probiotics by efficient vibration technology*” [3], where Microencapsulator provide by BÜCHI (Encapsulated B-390) was used.

**Table:** Specifications Table

Subject area	*Applied Microbiology and Biotechnology*
More specific subject area	*Microencapsulated probiotics with low and medium viscosity sodium alginate prepared by vibration technology.*
Type of data	*Graph*
How data was acquired	*Probiotic microcapsules were manufactured used a Microencapsulator BÜCHI (Encapsulated B-390) and viability was obtained after filtration and dissolution in sodium citrate to release microcapsules and count CFU in supernatant according Olivares* et al.(2017) [3].
Data format	*Raw and analyzed data.*
Experimental factors	*Viability of Lactobacillus casei expressed in* log *CFU/g spheres was determined according to Olivares* et al.(2017) [1].
Experimental features	*A factorial design (3*^*3*^*) was carried out with two microencapsulator variables: [frequency (*1000Hz, 3000Hz *and* 5000Hz*) and nozzle size (*450μm, 750μm *and* 1000μm*)] and three different alginate concentrations (1%, 2% and 3%).*
Data source location	*Valparaíso, Chile.*
Data accessibility	*Data are included in this article and supplemental file.*
Related research article	*Olivares, A., Silva, P., Altamirano, C. 2017. Microencapsulation of probiotics by efficient vibration technology. Journal of Microencapsulation, 34 (7): 667–674. doi.org/10.1080/02652048.2017.1390005*

**Table:** 

**Value of the Data** • The data present the importance of the alginate viscosity on the formation of gel beads for probiotics microencapsulation by vibration technology. • Help to identify the best microencapsulation variables. • Data of viability of *Lactobacillus casei* at low and medium viscosity sodium alginate presented can be used for choosing a correct gel matrix depending on its use. • The data can be easily replicated, mainly because the strain, matrix, and equipment used to prepare microcapsules are commercially available.

## 1 Data

**Fig. 1:**
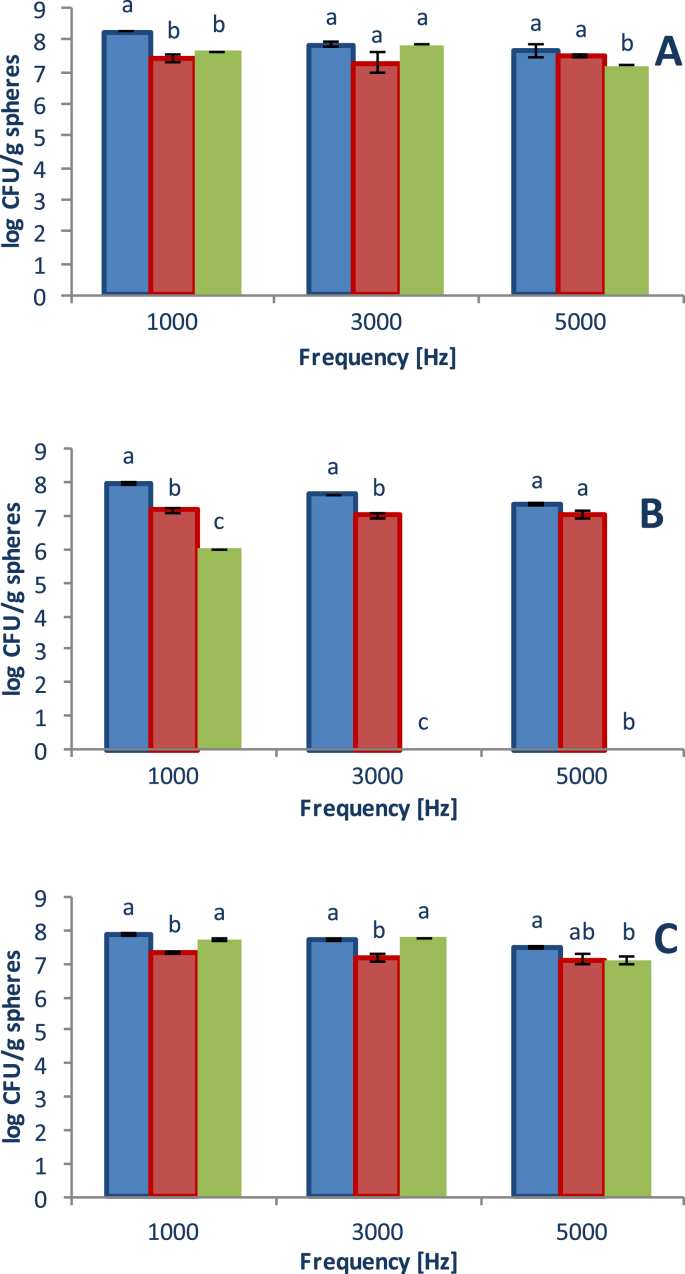
Viability of microencapsulated *Lactobacillus casei* at different frequency of microencapsulation and low viscosity alginate. **A**: alginate concentration 1%, **B**: alginate concentration 2%, **C**: alginate concentration 3%. (Nozzle size: : 450 μm, : 750 μm, : 1000 μm). Different letters mean a significant difference (*P* < 0.05) between the nozzle size in the same frequency group.

**Fig. 2:**
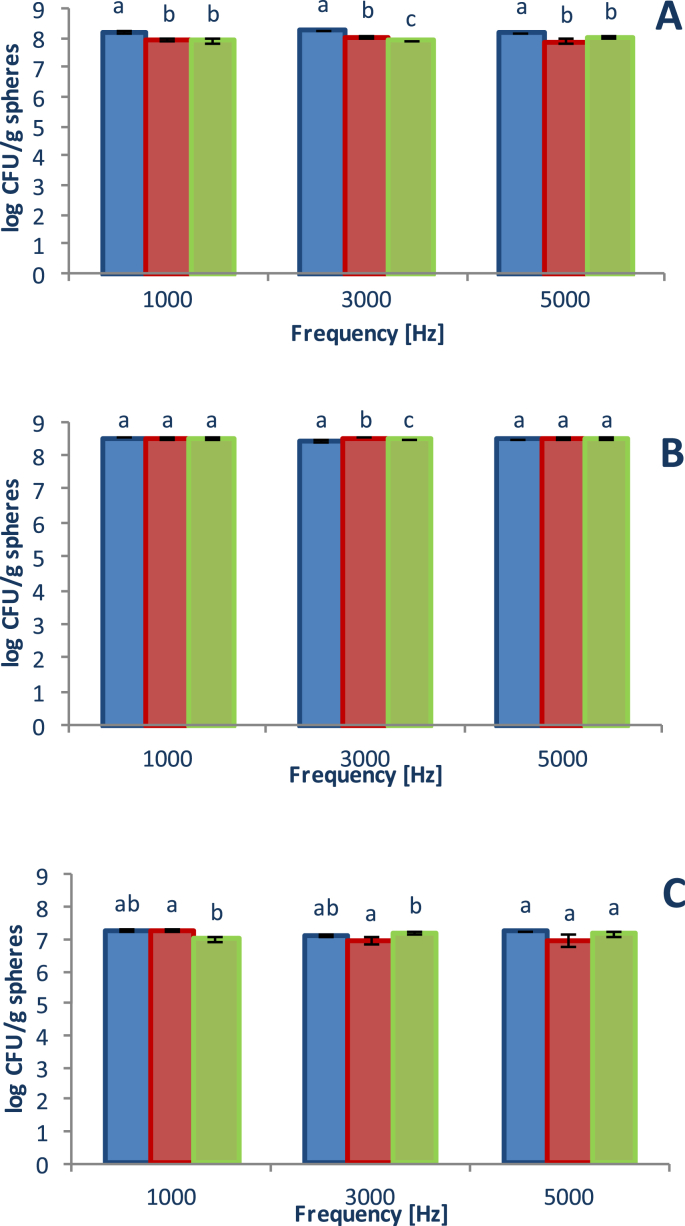
Viability of microencapsulated *Lactobacillus casei* at different frequency of microencapsulation and medium viscosity alginate. **A**: alginate concentration 1%, **B**: alginate concentration 2%, **C**: alginate concentration 3%. (Nozzle size: : 450 μm, : 750 μm, : 1000 μm). Different letters mean a significant difference (*P* < 0.05) between the nozzle size in the same frequency group.

Viability of microencapsulated *Lactobacillus casei* at a different frequency of microencapsulation and at different nozzle size 450 μm, 750 μm, 1000 μm and at low and medium viscosity sodium alginate concentration are shown in Fig. 1, Fig. 2, respectively. Data show 3 different alginate concentration, 1%, 2% and 3% (figures A, B, and C, respectively). The operational variables of the microencapsulation equipment can be modified to achieve a better target and these data represent the viability of *Lactobacillus casei* (log UFC/sphere). At 450 μm, viability range varied between 7.395 and 8.247 log CFU/spheres for three frequencies and low viscosity sodium alginate concentration, meanwhile at the same nozzle size range was between 7.131 and 8.530 log CFU/spheres for three frequencies medium viscosity sodium alginate concentration. At 750 μm, viability range varied between 7.019 and 7.469 log CFU/spheres for three frequencies and low viscosity sodium alginate concentration, meanwhile at the same nozzle size range was between 6.963 and 8.531 log CFU/spheres for three frequencies medium viscosity sodium alginate concentration. And at 1000 μm, viability range varied between 0.0 (at 3000 and 5000 Hz and concentration of 2%) and 7.845 log CFU/spheres for three frequencies and low viscosity sodium alginate concentration, meanwhile at the same nozzle size range was between 6.992 and 8.486 log CFU/spheres for three frequencies medium viscosity sodium alginate concentration. At low viscosity, the highest viability was obtained at a concentration of 3%, while at medium viscosity better viability was obtained at a level of 2% and for all the nozzles and frequencies studied, the viability was higher than 8.427 log CFU/spheres. The highest viability of the probiotics was at a 2% sodium alginate concentration (medium viscosity) and a frequency of 1000 Hz, and there was no significant difference (*P* < 0.05) between the size nozzle (the maximum viability value was 8.531 log UFC/g spheres at 450 μm and were the smallest beads size).

## 2 Experimental design, materials, and methods

### 2.1 Materials

*Lactobacillus casei* (DSM 20011) were supplied by DSMZ-Germany and used in lyophilized form according Olivares et al. [3].

Two kinds of alginic acid sodium salt from Sigma-Aldrich, St. Louis, MO, USA were used. Low viscosity (Sigma cod. A1112) with 4–12 cP and medium viscosity (Sigma cod. A2033) and grater than 2000 cP. A sophisticated microencapsulation technology developed by BÜCHI (Encapsulator B-390; CIENTEC Instrumentos Científicos, S.A. Chile) was used.

### 2.2 Microcapsules production

Each sodium alginate solution (low and medium viscosity) was prepared at double of its concentration and then mixed at 1:1 ratio with a *Lactobacillus casei* solution (prepared at 5g/L with lyophilized powder with more than 10 log CFU/g).

To form microcapsules, BÜCHI Encapsulator B-390 were used. Nozzle size and frequency were changed for each assay. Flow rate was constant (20 mL/min) and controlled by pump injector. Voltage of 250 V was used [2,3].

### 2.3 Microencapsulated cell count

The microcapsules were dissolved in 50mM sodium citrate (pH 7.5) to release *Lactobacillus* cells into the supernatant for counting according Olivares et al. [3].

### 2.4 Statistical analysis

The data are expressed as the mean ± standard deviation of triplicate experiments. The data was subjected to analysis of variance (ANOVA) to compare results and determine statistical significant difference (*P* < 0.05).
